# Clinical and Epidemiologic Characteristics of Norovirus GII.4 Sydney during Winter 2012–13 in Beijing, China following Its Global Emergence

**DOI:** 10.1371/journal.pone.0071483

**Published:** 2013-08-16

**Authors:** Huan Mai, Miao Jin, XiaoLin Guo, Jian Liu, Ning Liu, Xu Cong, Yan Gao, Lai Wei

**Affiliations:** 1 Peking University People’s Hospital, Peking University Hepatology Institute, Department of Infectious Diseases, Beijing, China; 2 Chinese Center for Disease Control and Prevention, National Institute for Viral Disease Control and Prevention, Beijing, China; 3 Peking University People’s Hospital, Department of Clinical Laboratory, Beijing, China; 4 Peking University People’s Hospital, Peking University Hepatology Institute, Beijing, China; Centers for Disease Control and Prevention, United States of America

## Abstract

**Background:**

Limited information is available on the molecular epidemiology of GII.4 Sydney-associated diarrhea in China in the winter of 2012–13 during the global epidemic associated with the emergence of GII.4 Sydney.

**Methods:**

Fecal specimens collected from 171 diarrhea outpatients (one from each) between late October 2012 and the middle of March 2013 were examined for NoV by reverse transcription-polymerase chain reaction and sequences corresponding to both the NoV partial polymerase and partial capsid regions were analyzed phylogenetically. Clinical characteristics of GII.4 Sydney cases versus other NoV-positive cases detected in a previous study were compared statistically.

**Results:**

Twenty-six (15.2%, 26/171) outpatients with diarrhea were infected with NoV. Twenty-two of the 26 (84.6%) identified NoV strains clustered into GII.4 Sydney. There was a significant difference in symptoms of fever (χ^2^, *P*<0.05 ), abdominal pain (χ^2^, *P*<0.05 ) and diarrhea frequency (Mann-Whitney U test, *P*<0.05) between the GII.4 Sydney case group and other NoV-positive case group.

**Conclusions:**

The new NoV variant, GII.4 Sydney, has been circulating in Beijing, China and became the predominant strain in the winter of 2012–13. GII.4 Sydney causes severe fever, abdominal pain and higher diarrhea frequency clinically compared to other NoV infections.

## Introduction

Diarrhea is a major cause of morbidity and mortality in all regions of the world and among all ages [1]. Noroviruses (NoVs) are well-documented as the most commonly detected pathogens in both sporadic cases and outbreaks of diarrhea worldwide [2], [3], [4], with a distinct seasonality linked to the winter months [5], [6]. NoV infections are associated with greater than 90% of non-bacterial gastroenteritis and approximately 50% of all epidemic gastroenteritis worldwide, and also account for 12% of mild and moderate cases of diarrhea irrespective of ages [4]. The highly infectious viruses usually cause self limiting disease, presenting with acute onset of nausea, vomiting, abdominal cramps, and watery diarrhea, which generally last approximately 48 h [7]. NoV infections are also called “gastric flu” not only due to similar seasonality and lack of effective therapeutics like influenza viruses, but also for its high infectivity and rapid evolution [3], [8].

NoVs belong to the family Caliciviridae. As is typical of positive-sense single-stranded RNA viruses, NoVs are highly diverse with approximately 46% nucleotide divergence across the genome among its five genogroups (GI–GV) [8]. GI and GII are involved in the majority of acute viral diarrhea cases in humans [5]. There is further diversity within each genogroup, resulting in the subdivision of GI and GII into up to 30 genotypes [9]. NoVs have caused at least four global epidemics of gastroenteritis over the past 15 years (1995–1996, 2002–2003, 2004–2005, 2006–2007), which were all associated with GII.4 (genogroup II genotype 4) [8], [10]. GII.4 is the primary genotype responsible for epidemics of acute gastroenteritis as well as sporadic cases in many countries. It is currently responsible for 60–80% of outbreaks worldwide with new variants emerging every 2 or 3 years [9]. According to a study by Bull et al. [11], the GII.4 lineage has a faster mutation rate and rate of evolution than other NoVs. Antigenic drift in response to human herd immunity and receptor switching are proposed as the major mechanisms driving the evolution of GII.4 [8].

In late 2012, surveillance systems in many countries (including the United Kingdom, the Netherlands, Japan, Australia, France, New Zealand and the United States) showed increased levels of NoV activity compared to previous seasons [12], [13]. Data from these countries indicated that this increase was associated with the emergence of a new variant of GII.4 named GII.4 Sydney [12], [13], [14]. Monitoring the emergence of a new GII.4 variant strain, which could display a different pathogenesis and virulence, is important for public health planning [15]. It is still unknown whether the increased global activity of the new GII.4 variant has extended to China and the new variant is circulating in our country. Molecular epidemiology and clinical characteristics of sporadic cases can reflect NoV activity and epidemic features to some extent. Peking University People’s Hospital (PKUPH), a teaching hospital affiliated with Peking University, receives over 2,000,000 outpatient cases annually. In this study, we conducted surveillance of diarrhea in PKUPH outpatients in the winter of 2012–13 to study the molecular epidemiology and clinical characteristics of GII.4 Sydney-associated diarrhea in Beijing, China.

## Materials and Methods

### Ethics Statement

This study was conducted in accordance with the ethical guidelines of the Declaration of Helsinki and in agreement with the Ethics Committee of PKUPH. We informed each potential subject of the details of our study, and once informed consent had been received, stool samples and medical data were collected and analyzed anonymously. We documented all information in approved information sheets instead of written consent forms. The whole process did not harm patients’ health, safety or privacy. This consent procedure has been approved by the Ethics Committee of PKUPH.

### Study Population

The study population included outpatients who sought medical attention in the Department of Infectious Diseases at PKUPH during the winter of 2012–13 (from late October 2012 to the middle of March 2013). Patients who presented with diarrhea defined as three or more loose stools within a 24 h period were included in this study. This time period was chosen as it was the season with the highest NoV incidence in northern China [6] and it was after the increased NoV activity was first reported by Japan, UK and many other countries [12].

### Fecal Specimens and Data Collection

One stool sample was collected from each outpatient who met the criteria. Data was collected at the time of medical consultation using a standardized questionnaire that included demographic information, epidemiological history and clinical observations (such as symptoms and temperature). We collected a total of 171 fecal specimens from 171 outpatients with diarrhea. Stool samples were immediately stored at −20°C until analysis.

The clinical information of NoV-positive outpatients in our previous study between October 2007 and September 2008 was used in statistical analysis along with GII.4 Sydney-positive outpatients [6]. The previous study was undertaken at the Department of Infectious Diseases in PKUPH and the same enrolment criteria, diarrhea definition and symptom questionnaire were used in both studies.

### Viral RNA Extraction and NoV RT-PCR

RNA was extracted from 140 µL of a 10% stool suspension in phosphate buffered saline (PBS, pH 7.4) using the QIAamp Viral RNA Mini Kit (Qiagen, Hilden, Germany) according to the manufacturer’s instructions. Reverse transcription was performed at 37°C for 50 min using random primers and Moloney murine leukemia virus reverse transcriptase (Invitrogen, Carlsbad, CA, USA). PCR was performed using primers P289 and P290 targeting the partial RNA-dependent RNA-polymerase (RdRp) region in ORF1 as previously described [6] and under the following conditions: 94°C for 5 min followed by 40 cycles of 94°C for 1 min, 52°C for 1 min and 20 s, 72°C for 1 min followed by a final extension step at 72°C for 10 min. The PCR products were stored −20°C until analysis.

The second RT-PCR targeted the partial capsid region in ORF2 using two primer pairs, GISKF-GISKR for GI strains and COG2F-G2SKR for GII strains as previously described [6]. RT was performed at 50°C for 1 h using SuperScript™ III reverse transcriptase (Invitrogen) and random primers. Conditions used for PCR were as follows: 94°C for 3 min followed by 35 cycles of 94°C for 30 s, 55°C for 30 s, 72°C for 60 s and a final extension step at 72°C for 7 min. The PCR products were stored at –20°C until analysis.

We obtained positive stool samples from the Chinese Center for Disease Control and Prevention (Chinese CDC), which were confirmed by RT-PCR and sequence analysis to be NoV GI and GII strains. RNA extracted from the stool samples were used as positive RT-PCR controls for each of the primer sets used.

### Sequence Analysis

The respective PCR products above were sequenced in both directions using an ABI3730XL DNA Analyzer (Applied Biosystems, Foster City, CA, USA). Nucleotide sequences were analyzed using CLUSTAL X (Version 1.83) followed by phylogenetic analysis using MEGA version 5.1. Statistically significant differences between inferred phylogenies were estimated using bootstrap analysis with 1,000 pseudoreplicate data sets. The phylogenetic trees were constructed using the maximum-likelihood method.

### Statistical Analyses

Data was entered into a database and analyzed using SPSS software version 16.0 (SPSS, Chicago, IL, USA). Continuously distributed variables were compared between GII.4 Sydney cases and other NoV-positive cases using the Mann-Whitney U test after normality test. Categorical variables were compared by Pearson χ^2^ test or continuity correction. One-way ordinal categorical variables were compared by the Mann-Whitney U test. *P* values <0.05 were considered statistically significant.

## Results and Discussion

### NoV Detection and Sequence Analysis

**Figure 1:**
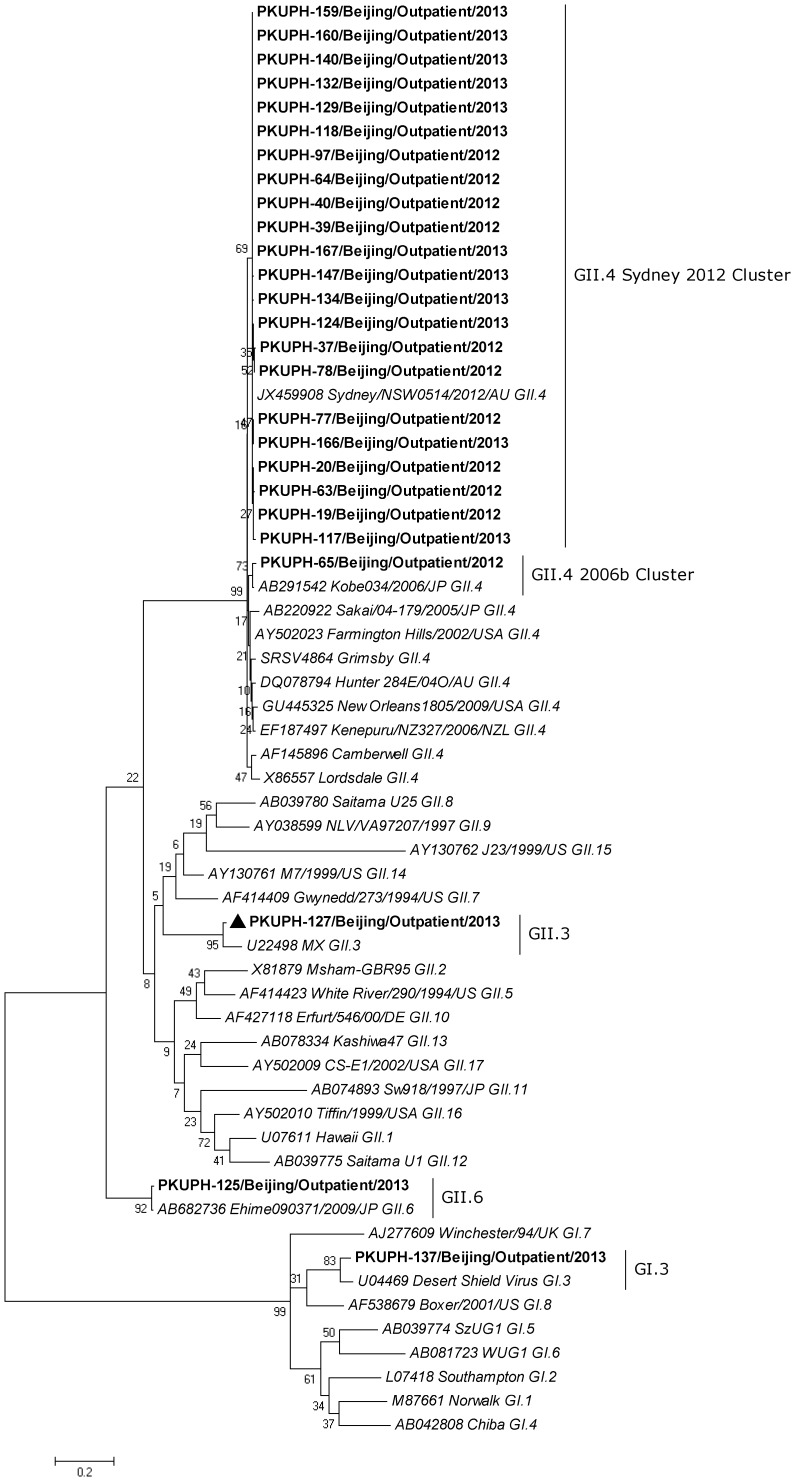
Phylogenetic analysis of the partial capsid (281 bp) sequence of the identified NoV strains in Beijing, China. The diagram shows the maximum likelihood analysis of the partial capsid sequence (ORF2) of NoV strains (n = 26, boldface) identified in stool samples from 26 outpatients presenting with diarrhea. Twenty-five (96.2%, 25/26) of 26 strains clustered with GII, in which 23 (88.5%, 23/26) clustered with the GII.4 genotype and the remaining one belonged to the GI.3 genotype. In total, 22 strains (84.6%, 22/26) clustered with GII.4 Sydney and displayed 100% amino acid sequence identity with GII.4 Sydney variant (Accession No. JX459908). The maximum-likelihood phylogenetic tree was generated using the software program MEGA, version 5.1. A Kimura 2-parameter model was used for nucleotide substitution, and substitution rates were assumed to be gamma distributed with invariant sites (G+I). The final tree was optimized using the heuristic nearest-neighbor-interchange (NNI) method. The numbers on each branch indicate the bootstrap values. The reference strains of GI (GI.1–GI.8) and GII (GII.1–GII.17) are shown in italics and GenBank accession numbers are included. The clustering is based on the classification of Zheng et al. [24].

We collected 171 fecal specimens from 171 outpatients from late October 2012 to the middle of March 2013. NoVs were detected from stool specimens of 26 (15.2%, 26/171) outpatients by RT-PCR. Partial capsid sequences (ORF2) of 26 strains were sequenced. Sequence analysis of the partial capsid region (Figure 1) shows that 25 strains from 26 (96.2%, 25/26) patients belonged to GII and the remaining strain (PKUPH-137/Outpatient/Beijing/2013) clustered with the GI.3 genotype. Twenty-three strains (88.5%, 23/26) clustered with the GII.4 genotype, in which 22 strains (84.6%, 22/26) grouped with GII.4 Sydney and displayed 100% amino acid sequence identity with GII.4 Sydney variant (Accession No. JX459908). Another GII.4 strain (PKUPH-65/Outpatient/Beijing/2012) clustered into the 2006b variant that was the predominant strain in China in past years [6], [16], [17], [18] and shared 100% homology with the 2006b variant (Accession No. AB291542). The remaining two GII strains clustered into GII.3 (PKUPH-127/Outpatient/Beijing/2013) and GII.6 (PKUPH-125/Outpatient/Beijing/2013).

**Figure 2:**
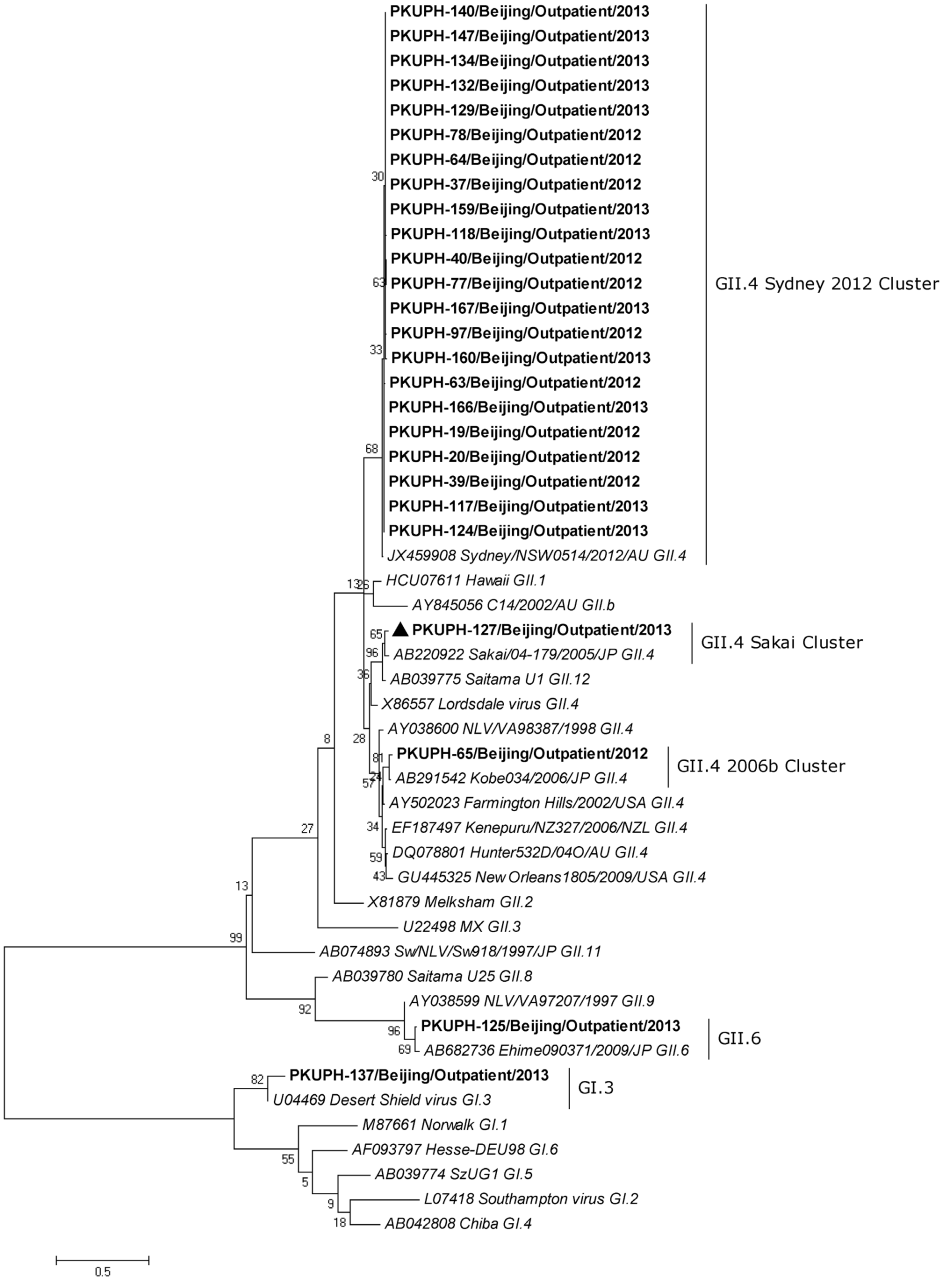
Phylogenetic analysis of the partial RdRp (273 bp) sequence of the identified NoV strains in Beijing, China. All the 26 RdRp sequences of NoV strains were obtained by RT-PCR amplification. The NoV strain (PKUPH-127/Outpatient) with a distinct genotype following the analysis of capsid regions that has conflicting RdRp genotypes is marked by a solid triangle. The remaining 25 NoV strains displayed the same genotypes based on capsid and RdRp regions. A Kimura 2-parameter model in the maximum-likelihood phylogenetic tree was used for nucleotide substitution, and substitution rates were assumed to be gamma distributed (G). The final tree was optimized using the heuristic nearest-neighbor-interchange (NNI) method. The numbers on each branch indicate the bootstrap values. The reference strains of GI (GI.1–GI.6) and GII (GII.1–GII.12 except GII.5, GII.7 and GII.10) are depicted in italics and GenBank accession numbers are included. The clustering is based on the classification of Zheng et al. [24].

Phylogenetic analysis of these capsid and RdRp sequences demonstrated that 25 out of the 26 NoV strains revealed the same genotypes based on capsid and RdRp regions (Figures 1 and 2). Only one strain (PKUPH-127/Outpatient/Beijing/2013) showed an inconsistent genotype of a GII.4 Sakai polymerase and a GII.3 capsid (Figures 1 and 2), which indicated a potential NoV recombinant as reported previously [19].

Nucleotide sequences obtained from this study were deposited in GenBank under accession numbers KC709581-KC709598 and KC970246-KC970253 (26 capsid sequences) and KC709608-KC709625 and KC970258-KC970265 (26 RdRp sequences).

### GII.4 Sydney Variant is the Predominant Strain of NoV in Beijing

Since 2006, many molecular epidemiological NoV surveys have demonstrated that NoVs are among the primary pathogenic causes of sporadic and epidemic diarrhea in China, and GII.4 2006b was the most predominant strain circulating in both children and adults [6], [16], [17], [18], [20]. The weekly distribution of sample collections, NoV detection and genotype in this study is shown in Table 1. The GII.4 Sydney variant was confirmed in all the weeks in which NoV was detected. The high rate (84.6%, 22/26) of GII.4 Sydney strains in NoV-associated sporadic diarrhea cases in our study indicates the new variant was circulating as the predominant strain in Beijing, China, in the winter of 2012–13. This was the first epidemiological study to report on the dominance of the new GII.4 Sydney variant circulating in China. The identification of novel GII.4 variants may also lead to an improved ability to predict increases in NoV activity [7].

**Table 1:** Weekly distribution of sample collections, NoV detection and genotype from week 43, 2012 to week 11, 2013.

Week	Total	NoV cases	GII.4 Sydney cases	GII.4 2006bcases	RecombinantGII.3/GII.4cases	GII.6 cases	GI.3 cases
43	6	0	0	0	0	0	0
44	14	2	2	0	0	0	0
45	12	0	0	0	0	0	0
46	12	3	3	0	0	0	0
47	9	0	0	0	0	0	0
48	21	3	2	1	0	0	0
49	8	2	2	0	0	0	0
50	9	0	0	0	0	0	0
51	10	1	1	0	0	0	0
52	9	0	0	0	0	0	0
1	6	0	0	0	0	0	0
2	7	2	2	0	0	0	0
3	10	5	3	0	1	1	0
4	13	3	2	0	0	0	1
5	1	0	0	0	0	0	0
6	1	0	0	0	0	0	0
7^a^	0	0	0	0	0	0	0
8	11	1	1	0	0	0	0
9	2	1	1	0	0	0	0
10	8	3	3	0	0	0	0
11	2	0	0	0	0	0	0

**NOTE.** ^a^We did not collect samples during week 7 because of the Spring Festival.

Many countries in Europe, as well as Japan and the United States have recently reported an increased NoV activity caused by GII.4 Sydney. The UK Health Protection Agency (HPA) reported that there were 4,407 laboratory-confirmed cases (from week 27, 2012 to week 01, 2013) in England and Wales, which is 56 percent higher than reported at this point last year [21]. Increases in the incidences of NoV-related gastroenteritis and deaths in the elderly were also described in Japan [12]. Similarly, increases have been noted in Australia, France, New Zealand and the United States [12], [13]. CDC in the United States reported that GII.4 Sydney was responsible for 53% of the NoV outbreaks and noted a statistically significant increase in the proportion of outbreaks caused by GII.4 Sydney from September-December 2012 [13]. In a recent study from Denmark, Fonager et al. found that GII.4 Sydney strain was sporadically detected since January 2012 and rapidly emerged as the dominant variant after October 2012 [22]. In this study, the detection rate (15.2%, 26/171) of NoV in stool samples in sporadic patients is slightly higher than the rate (11.9%, 48/403) we previously observed between October 2007 and September 2008 [6]. The selection of samples and the research period might be the primary reasons for this. It is difficult to estimate the incidence of GII.4 Sydney in China precisely, due to the scale of our study. More studies of other areas in China are necessary to monitor the nationwide prevalence and epidemiology of the new variant.

### Clinical Characteristics of GII.4 Sydney- positive Outpatients

The demographics and clinical characteristics of the 22 GII.4 Sydney-positive outpatients are shown in Table 2. For the limited number of other NoV-positive cases in this study, we compared the demographic and clinical characteristics of GII.4 Sydney-positive outpatients with the information obtained from 48 NoV-positive outpatients identified in our previous study between October 2007 and September 2008 [6] (Table 2). Of the 48 previously-isolated NoV strains which did not cluster with GII.4 Sydney, 44 (91.7%, 44/48) strains belonged to GII and 37 (77.1%, 37/48) strains clustered with the GII.4 2006b strain, based on the partial RdRp region.

**Table 2:** Comparison of the clinical characteristics of GII.4 Sydney with other NoV-positive outpatients in Beijing, China.

Variables	GII.4 Sydney case (n = 22)	Other NoV-positive case (n = 48)^a^	*P-*Value
Age (years)	31 (25.75–59.75)	48 (29–64.5)	0.168
Male sex	9 (40.9%)	23 (47.9%)	0.385
Nausea^bc^	15 (71.4%)		
Vomiting^c^	9 (42.9%)	13 (27.1%)	0.267
Fever (≥37.3°C)^c^	6 (28.6%)	1 (2.1%)	0.004
Mild (37.3–38.0°C)	5	1	
Moderate (38.1–39.0°C)	1	0	
Abdominal pain^c^	13 (61.9%)	13 (27.1%)	0.006
Frequency of diarrhea^c^	10 (8–10)	7 (5–10)	0.011
Dehydration^c^			0.609
No	15	36	
Mild	4	12	
Moderate	2	0	

**NOTE.** Values are median (IQR) or n (%) of patients unless otherwise stated.

a The information for this group was obtained from NoV-positive outpatients in our previous study between October 2007 and September 2008.

b The information from other NoV-positive case group was missing.

c One participant in GII.4 Sydney case group was missing information on symptoms.

Among 22 patients infected with GII.4 Sydney, 71.4% reported nausea, 42.9% vomiting, 28.6% fever and 61.9% abdominal pain. The median diarrhea frequency was 10 [Interquartile range (IQR) 8–10]. There were six GII.4 Sydney-positive cases presenting with dehydration (four mild and two moderate). Only one patient presented with mild respiratory symptoms (data not shown). We inquired the epidemiological history of 15 GII.4 Sydney-positive patients (data not shown) and found that most patients had not recently travelled, with the exception of one patient on a business trip from Jilin Province (more than 1000 kilometers northeast of Beijing), which suggests that the new variant was circulating in Beijing prior to the winter studied in this report.

Statistical analyses showed that there was a significant difference in patients presenting with symptoms of fever, abdominal pain and increased diarrhea frequency between the GII.4 Sydney case group and the other NoV-positive case group. Patients infected with GII.4 Sydney were more likely to present with symptoms of fever (χ^2^, *P*<0.05 ) and abdominal pain (χ^2^, *P*<0.05 ). Table 2 shows that of six GII.4 Sydney-positive patients presenting with fever, five experienced mild fever (37.3–38°C) while the remaining one experienced moderate fever (38.1–39°C), which suggests that the degree of fever tends to be mild. GII.4 Sydney-positive patients experienced a higher frequency of diarrhea than other NoV-positive patients (Mann-Whitney U test, *P*<0.05).

With the limited sample size, it is difficult to conclude that patients infected with GII.4 Sydney present with more common and severe symptoms as compared with other NoV-positive patients. There are few reports on the clinical presentation of GII.4 Sydney infections from other countries. More clinical studies from other countries are necessary to conclusively demonstrate the clinical characteristics of GII.4 Sydney infection.

### Conclusion

Our data indicate that the new variant GII.4 Sydney that was associated with an increase in worldwide NoV activity in late 2012 has been circulating in Beijing, China, and was the predominant of NoV in the winter of 2012–13. Patients with sporadic infections with GII.4 Sydney were more likely to present with symptoms of fever (χ^2^, *P*<0.05 ) and abdominal pain (χ^2^, *P*<0.05 ) and have higher diarrhea frequency (Mann-Whitney U test, *P*<0.05) compared with other NoV-positive cases. [23].
